# Long-term recurrence of Dupuytren’s disease treated with clostridium histolitycum collagenase. Surgical treatment and anatomopathological study

**DOI:** 10.1007/s00402-024-05320-7

**Published:** 2024-04-23

**Authors:** C. Simón-Pérez, J. I. Rodríguez-Mateos, I. Aguado Maestro, M. Alvarez-Quiñones, E. Simon-Perez, M. A. Martín-Ferrero

**Affiliations:** 1 Discipline of Orthopaedics, University of Valladolid, Valladolid, Spain; 2 Department of Plastic Surgery, Hospital Universitario Rio Hortega, Valladolid, Spain; 3 Department of Pathological Anatomy, Hospital Clínico Universitario, Valladolid, Spain; 4 DP Recoletas Felipe II Hospital C/ Felipe II, Valladolid, 9 47003 Spain; 5 Hospital Clínico Universitario, Avenida Ramón y Cajal, s/n, Valladolid, 47005 Spain

**Keywords:** Collagenase, *Clostridium histolyticum*, Dupuytren recurrence, Fasciectomy

## Abstract

**Objective:**

To present the functional results obtained and the possible surgical difficulties after the surgical treatment of Dupuytren’s disease (DD) recurrence in patients previously treated with *Clostridium histolyticum* (CCH) collagenase.

**Materials and methods:**

In this prospective study, 178 patients with DD were treated with CCH from 2011 to 2018; During long-term postoperative follow-up, 34 patients (19.1%) had recurrence of DD. In all patients injected in the IFP the disease recurred; In patients injected in the MCP, recurrence was highest in grade III and IV of the Tubiana classification, with involvement of the 5th finger and the two-finger Y-chord. Fourteen patients (7,8%) required surgery by partial selective fasciectomy due to recurrence of cord DD infiltration. The clinical and functional results of the patients, the difficulty of the surgical technique and the anatomopathological analysis of the infiltrated cords were evaluated in comparison with those of cords and patients who had had no previous CCH treatment.

**Results:**

In all patients, cord rupture was achieved after injection, reducing joint contracture. In 14 patients, we observed during the follow-up the existence of DD recurrence that required surgical treatment by selective partial fasciectomy. There were no major difficulties in surgery and good clinical and functional results at 6 months of follow-up. The anatomopathological study of the resected tissue did not present histological alterations with respect to the samples obtained from patients initially treated by selective partial fasciectomy.

**Conclusions:**

Selective fasciectomy after CCH injection does not lead to important operative difficulties, as long as the CCH injection is performed according to the recommendations. There were no histological changes in the tissue after CCH injection.

**Level of evidence:**

III.

**Supplementary Information:**

The online version contains supplementary material available at 10.1007/s00402-024-05320-7.

## Introduction

Dupuytren’s disease (DD) is a progressive fibroproliferative disorder characterized by the development of collagen nodules and cords at the superficial palmar aponeurosis level that causes progressive finger closure [1].

There is no cure definitive for DD, and recurrence and progression of this disease over time are considered unavoidable [1].

Surgery is indicated in DD patients when they have joint contracture of more than 30° at the metacarpo-phalangeal joint (MCP) or any degree of joint contracture at the interphalangeal joint (PIP) [2, 3].

Therapeutic alternatives are selective partial fasciectomy, the most commonly used; percutaneous fasciotomy, with recurrence rates higher than selective partial fasciectomy; early-stage radiation [4, 5], and collagenase from *Clostridium histolyticum* (CCH), the only pharmacological treatment approved for the treatment of DD [6, 7]. This treatment has been established as an effective and safe modality for the treatment of DD because it reduces the degree of contracture of the affected fingers [7, 8].

DD recurrence is frequent after surgical treatment [9], especially in young patients [1]. The recurrence rate after surgery is highly variable, from 0 to 85%, depending on the patient characteristics, the disease and the type of surgery performed [1].

This variability in the DD recurrence rate is due to the lack of consensus and subjectivity in the definition of recurrence [1, 10]. Felici et al. (2014) defined recurrence as the existence of a passive extension deficit of more than 20° in at least one of the treated joints in the presence of a palpable cord, compared with the results obtained 6–12 weeks before [11].

The surgical treatment of DD has significant morbidity, with an index of complications of approximately 17%, including skin problems, haematoma, nerve damage, and reflex sympathetic dystrophy (CRPS) [12]. In some published studies, the complication index increased to 39% in both the surgical and postoperative stages [13].

There are publications on DD recurrence or progression after CCH injection, the most important factors being age under 60 years, the severity of the disease and the involvement of PIP [14, 15].

Therapeutic alternatives for treating DD recurrence or progression after CCH injection are either reinjection of the CCH or selective palmar fasciectomy on the injected cord [15].

The aim of this study was to evaluate the influence of CCH injection on rescue surgery and the histological changes in the infiltrated cord.

## Materials and methods

In a prospective, protocolled study, one hundred seventy-eight patients affected by DD were treated with CCH from 2011 to 2018. Fourteen patients were identified who required surgery by partial selective fasciectomy due to recurrence of cord infiltration after treatment of the disease with CCH.

The inclusion criteria were patients with DD who had previously been injected with CCH toxin, a DD recurrence in the area previously inoculated with a palpable cord, and a joint contracture of at least 30°.

### Operative technique

All patients were treated by outpatient surgery and were administered a CCH injection, taking into account the specific doses of both solvent and collagenase required, depending on the joints to be treated, according to the recommendations of the product (Xiapex®). Finger extension and cord rupture were performed after 24–36 h in an outpatient operating room with local-regional anaesthesia or sedation.

The salvage technique after DD recurrence was performed as outpatient surgery with local-regional anaesthesia of the affected limb and local ischaemia. The surgical technique consisted of selective partial fasciectomy of the affected area and zetaplasties, according to the usual technique. We performed anatomical-pathological analysis of the sample obtained.

### Assessment

Follow-ups were performed first weekly, then every 2 weeks, every month, every 3 months and every 1 year. In the first reviews, the presence of local complications (haematoma, skin dehiscence, vasculo-nervous lesions, etc.), decreased joint contracture and increased range of motion were evaluated.

Measurements were performed with a standard goniometer assessing joint contracture and range of motion according to the criteria of the International Federation of Societies for Surgery of the Hand (IFSSH).

Defined recurrence as the existence of a passive extension deficit of more than 20° in at least one of the treated joints in the presence of a palpable cord, compared with the results obtained 6–12 weeks before [11].

### Statistical study

Quantitative variables are presented with the mean and standard deviation and qualitative variables according to their frequency distribution.

Using Pearson’s Chi-square test, we analysed the association of the qualitative variables. In the event that the number of cells with expected values less than 5 was greater than 20%, we used Fisher’s exact test or the Likelihood Ratio test for variables with more than two categories. We analysed time to recurrence using Kaplan Meier analysis and mortality tables.

We will analyse the data with the statistical software IBM SPSS Statistics version 20.0 for Windows. Values of *p* < 0.05 will be considered statistically significant.

All patients signed a treatment-specific consent prior to the injection of CCH. The study was approved by the Hospital’s Clinical Research Ethics Committee (PI 17-548- CINV 16–56).

## Results

### Clinical characteristics and immediate postoperative follow-up

Of the 178 DD patients, 163 were male (91.6%) and 15 were female (8.4%). They were aged between 45 and 89 years, with a mean of 69.8 years; only 12 patients were under 60 years of age (6.7%).

101 patients (were injected in 4º finger (56,7%), 64 patients 5º finger (36,9%) and 13 patients cord Y affecting two fingers. (7,3%)

The classification of patients according to the severity of the disease, the Tubiana classification, is shown in Table 1.

**Table 1:** The classification of patients according to the severity of the disease, the Tubiana classification

Grades	Angles	Patients	Percentage
I	1–45º	31	17,4%
II	46–90º	62	34,8%
III	91–135º	58	32,6%
IV	> 135º	27	15,2%
Total		178	100,0%

A single injection of CCH was given to all patients; 164 patients (92.1%) were injected in a palpable cord at the MCP level, 151 one finger cord and 13 two finger Y-cord, and 14 patients (7.9%) were injected into a cord at the PIP level.

In all patients, cord rupture was achieved in the surgical act, increasing the range of motion. In one 70-year-old patient, CCH injection was probably not effective due to poor inoculation, and we performed a percutaneous needle fasciotomy to achieve complete cord rupture.

During long-term postoperative follow-up, 34 patients (19.1%) had recurrence of DD, with a mean age of 67.28 years, slightly lower than the mean age of patients without recurrence (69.9 years). In all patients injected in the IFP, the disease recurred; The highest recurrence rates were recorded in 5 patients with involvement of the Y-chord of two fingers (35.7%) and in 21 patients with involvement of the 5th finger (32.8%). This result is statistically significant.

Disease severity (Tubiana classification) and recurrence rates during follow-up were higher in patients with Tubiana grade IV 20 patients (74.1%) and grade III 13 patients (22.4%) being statistically significant. In grade II there was no recurrence in any patient and in one patient with grade I (3.2%).

During long-term postoperative follow-up, only 14 patients (7.8%) presented DD recurrence that required surgical operation, which was done by selective fasciectomy and zetaplasty.

All patients were male and right-handed, and the hand affected was the right in 8 patients. The mean age of 62.5 years (55–70).

All patients were previously punctured at the level of the MCP, six patients at the level of the 4th finger, seven patients at the level of the 5th finger and one patient at the level of a Y-cord between the 4th and 5th fingers.

The follow-up period between the CCH injection and the intervention was between 1 and 10 years with a mean of 5,4 years.

DD recurrence caused less joint contracture than before the puncture of the CCH at the MCP joint in thirteen patients; one patient had a somewhat greater progression, probably due to the difficulty of inoculating the CCH when the cord was not clearly palpated; in twelve patients, the progression of DD at the PIP joint of the treated finger was greater than before the CCH injection (Table 2).

**Table 2:** Degree of joint contracture of MCP and IFP of the hand prior to CCH injection (Initial) and prior to selective palmar fasciectomy (Recurrence**)**

Patients	MCP pre CCH	MCP pre F	IFP pre CCH	IFP pre F
1	75	45	50	85
2	25	30	0	10
3	25	10	0	60
4	30	30	70	35
5	80	65	0	30
6	90	75	10	15
7	95	55	10	20
8	50	40	10	30
9	65	50	10	15
10	95	70	0	30
11	45	35	10	40
12	70	35	35	30
13	90	60	20	10
14	85	45	10	25
	65,7	46,1	16,8	31,1

**Fig. 1:**
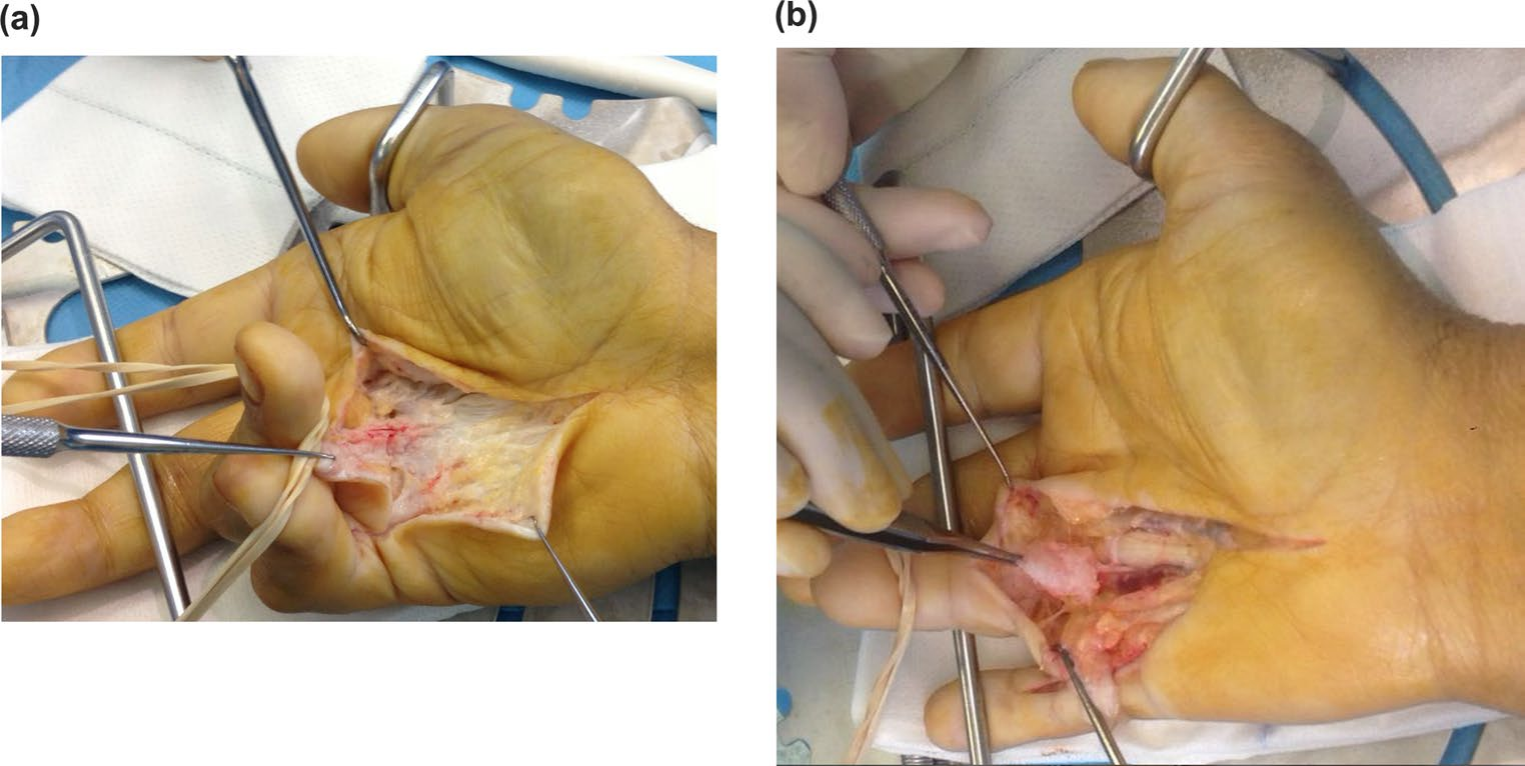
Selective fasciectomy 50 months after CCH puncture. Macroscopic aspect

During surgery in these patients, a fibrosis zone was observed at the puncture site level, but it did not create any difficulties for surgery (Fig. 1).

**Fig. 2:**
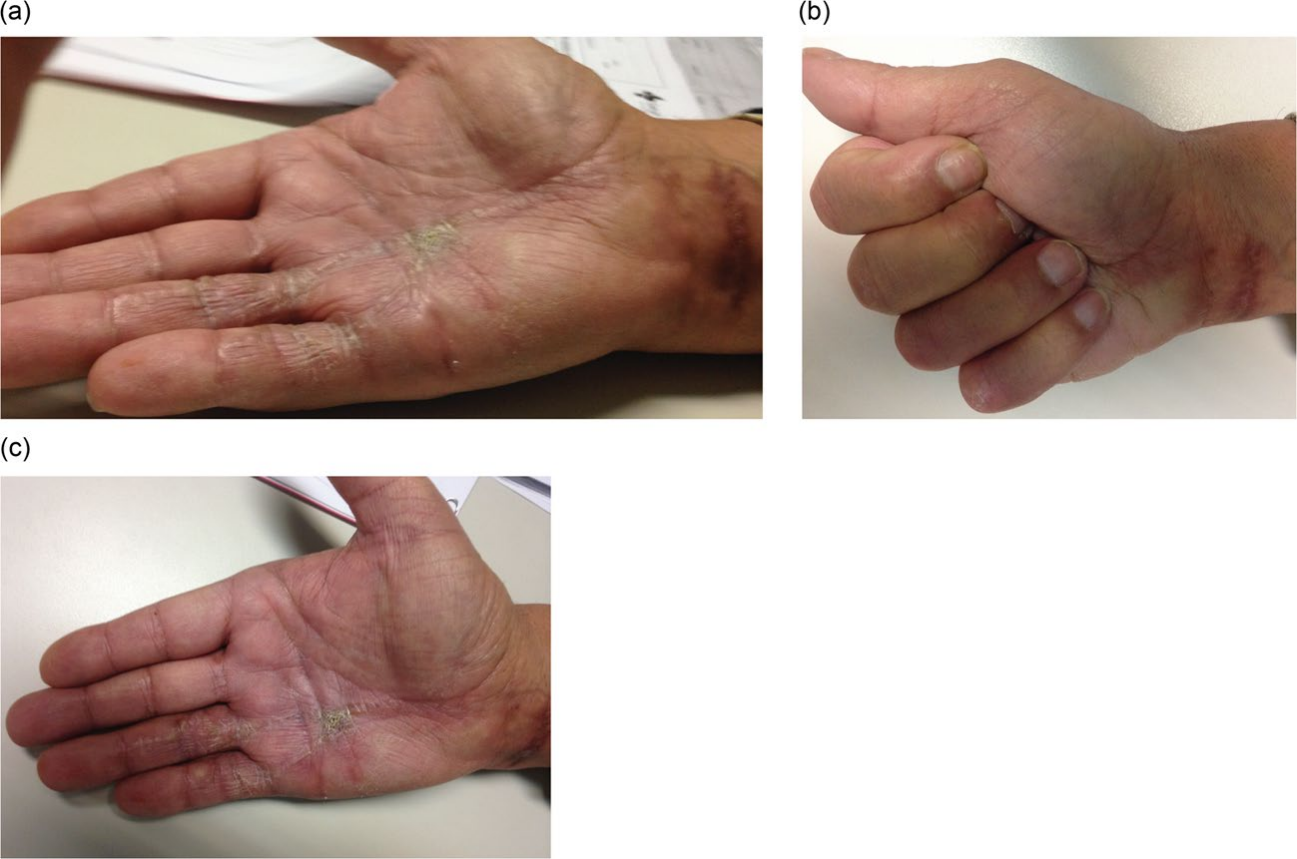
Functional and skin condition of the hand at the 1.5-month postoperative follow-up

In the immediate postoperative period, eight of these operated patients had skin dehiscence in the old puncture area treated by local cures, without problems of secondary scarring. No major local complications were observed (Fig. 2). All patients underwent postoperative rehabilitation with satisfactory functional recovery (Table 3).

**Table 3:** Mobility arch achieved after selective palmar fasciectomy compared to the degree of joint contracture prior to surgery

Patients	Mobility prior to surgery	Mobility 6 m after selective palmar fasciectomy
1	50	170
2	140	180
3	110	170
4	105	175
5	85	160
6	90	170
7	105	165
8	110	170
9	115	175
10	80	160
11	105	160
12	115	165
13	110	170
14	110	170

The macroscopic aspect of the material removed was not significantly modified compared with the material removed in patients with DD who had selective palmar fasciectomy as their first treatment option, except for an area of fibrosis in the previous puncture site.

**Fig. 3:**
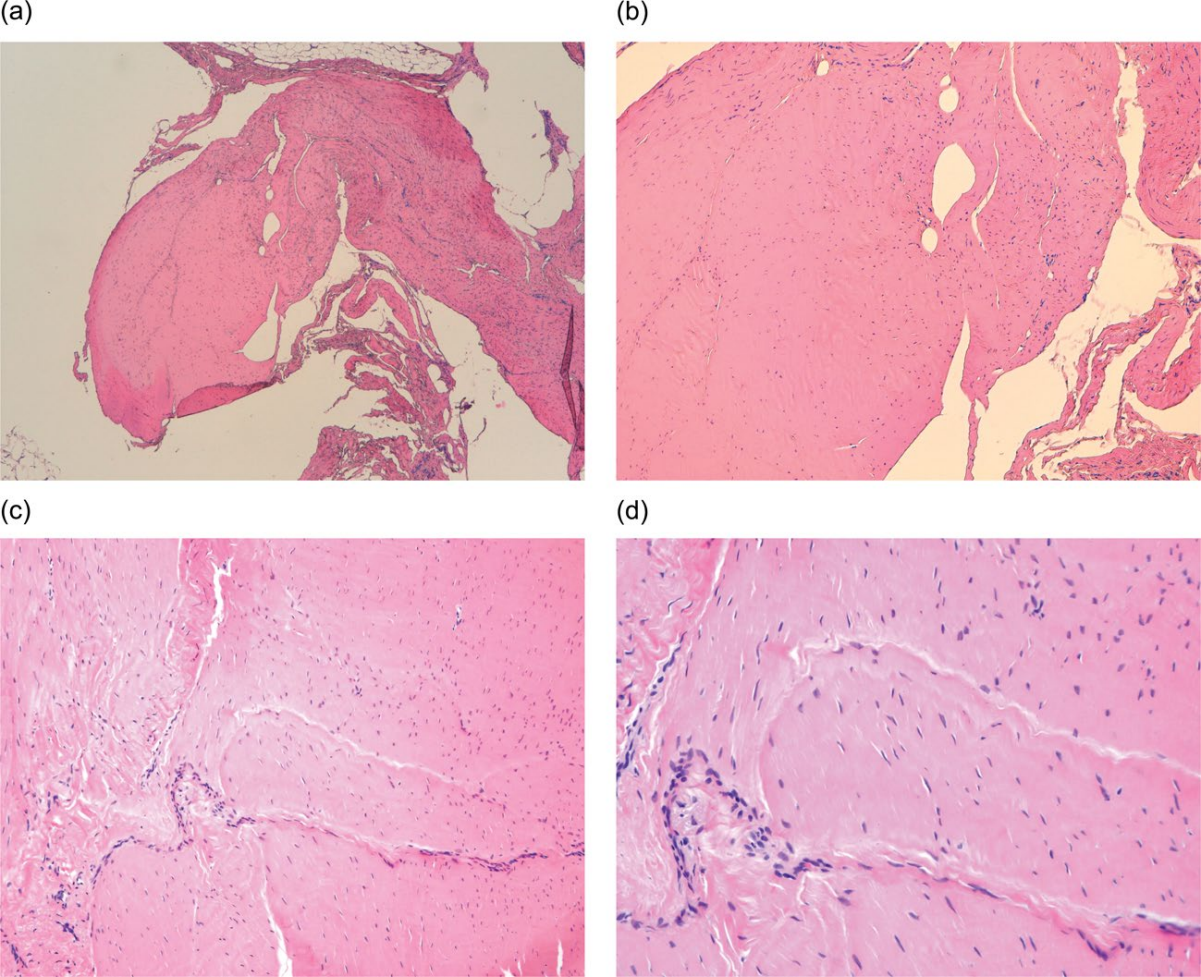
Microscopic aspect of the tissue removed after selective fasciectomy in a patient with previous CCH inoculation. **A**) General appearance of one of the histological sections of the biopsy, showing fibrosis of the palmar fascia (HE 40x). **B**) 100X: Histological image of the lesion, with predominance of dense fibrosis over a moderate number of fibroblasts (HE 100X). **C**) 200X and **D**) 400X: Dense fibrosis with extensive hyalinization, although collagen fibres are also observed (HE 200x and 400x)

**Fig. 4:**
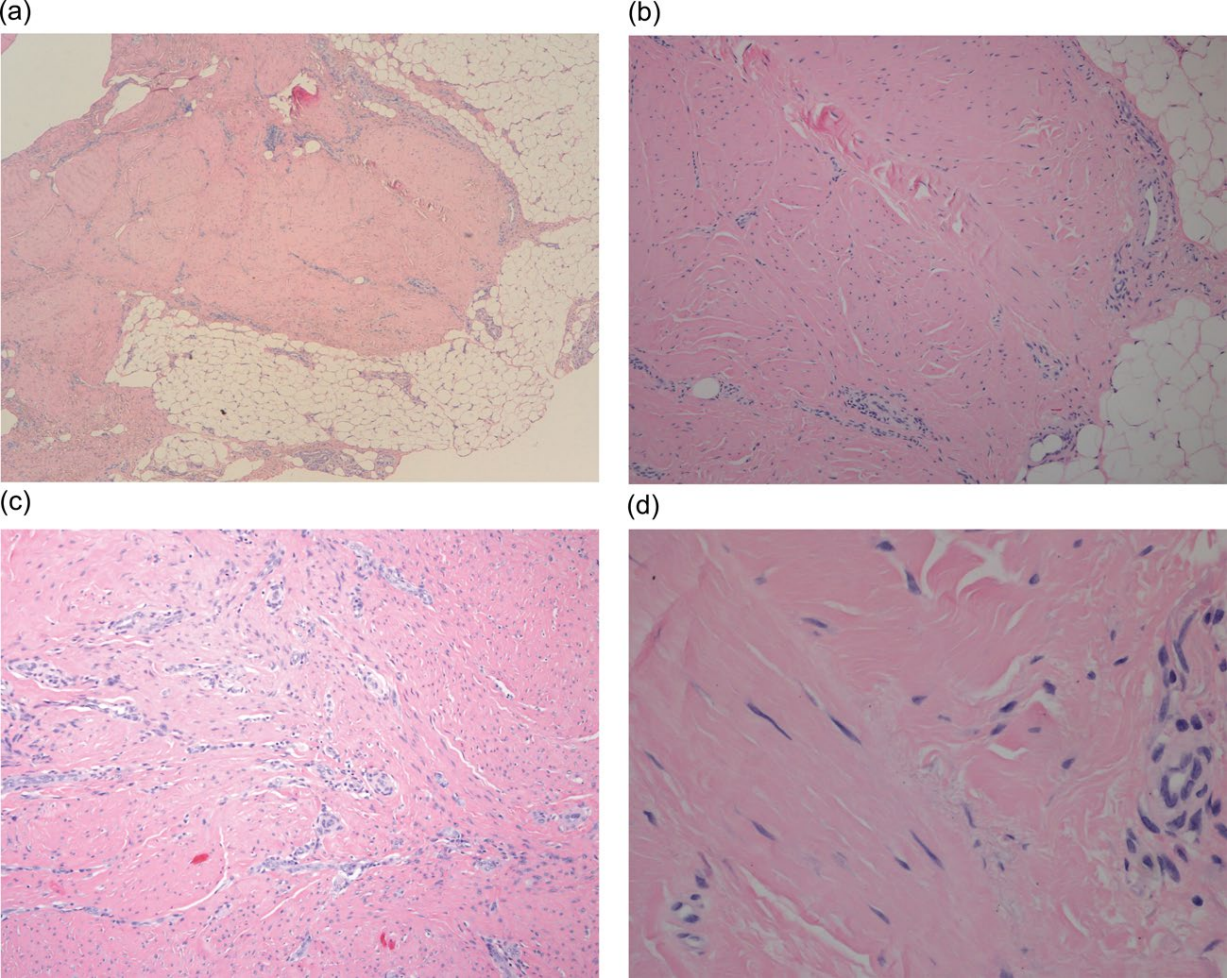
Microscopic histological aspect of tissue removed in a patient with DD initially operated on by selective fasciectomy. **A**) 40X: General aspect of the biopsied tissue, showing fibrosis of the palmar fascia (HE 40x). **B**) 100X: Histological image of the lesion, with predominance of dense fibrosis over a moderate number of fibroblasts (HE 100X). **C**) 200X and **D**) 400X. At medium and high magnification, abundant collagen fibres are observed, which predominate over fibroblasts (HE 200x and 400x)

The histological study was carried out using haematoxylin-eosin staining of the samples of the excised tissue in patients who had previously been injected with CCH, and advanced fibrosis was observed in all of them, with a fasciculate-nodular disposition, scarce-moderate fibroblastic cellularity, and collagen fibre production (Fig. 3). These samples were compared with samples of tissue removed from patients with DD initially treated by selective fasciectomy, and no difference was observed between the histological lesions of the cases treated conventionally and those that received injection by CCH (Fig. 4).

## Discussion

The therapeutic options for DD treatment are multiple depending on the patient’s characteristics and the disease itself, through surgical treatment: fasciectomies, dermofasciectomies, fasciotomies, and pharmacological treatment, namely, local injection of CCH [16].

CCH is the first pharmacological option approved for the treatment of adult patients with DD, and it avoids the complications associated with surgery [17]. Several published studies confirm the efficacy of CCH in DD, with clinical and functional improvement and rapid recovery in all patients in whom it has been administered [1, 8, 12, 14, 15].

We have observed the safety of CCH administration, since there have been few local complications, all of slight severity and easy resolution; we have not observed any serious local complications (tendon, vascular or nerve damage) or general complications. However, there are publications documenting serious complications such as tendons ruptured by intratendinous puncture and important cutaneous necrosis [18–20]. Compared with surgery, which has a complication rate ranging between 4% and 39% [13], CCH is a treatment with fewer complications and is also less invasive and therefore a good alternative to DD treatment, especially in elderly patients with multiple associated pathologies who are limited in daily activities, in whom surgery leads to an increase in the local and general complications rate [14, 21].

It is important to differentiate between recurrence and progression in DD, especially in patients treated with CCH in a single joint, to determine the actual efficacy of drug treatment, since DD is a progressive disease that can affect other joints over time regardless of treatment [14].

As other publications, we observed in our study that recurrence is higher and statistically significant, at the IFP level, when two fingers are affected, the greater the severity of the disease and in the 5th finger [8, 14, 15].

Patients who have suffered DD recurrence or progression during follow-up are highly satisfied because the degree of retraction of the affected finger is lesser than before the injection in most patients [14, 15].

Only fourteen patients (7.8%) in our study underwent a salvage palmar fasciectomy; compared with that in other publications [15], this number of patients is low, probably because the mean age of the patients in our study was higher, and only 12 patients were under 60 years of age (6.7%). It has been published [14] that younger patients were more likely to have a recurrence, and ten of the patients we operated on were under 65 years of age; Patient 70 years old, with involvement of the MCP and the PIP, it was necessary to perform a percutaneous needle fasciotomy at the MCP level to complete the cord rupture, with recurrence due to inoculation of the defective CCH.

As reported in other studies, in twelve patients the progression of DD in the PIP joint of the treated finger was greater than before the CCH injection [14, 15].

In the patients who required surgery, we observed scarring fibrosis in the area where the product was injected, without difficulty in the surgical technique, taking into account what has been published in other studies [21, 22].

A significant percentage of patients, 57.1%, presented skin dehiscence in the area of the old puncture, which we treated with local dressings without problems for secondary healing, and no major local complications were observed.

The histological study using haematoxylin-eosin staining of samples of removed tissue from patients who had previously been injected with CCH was compared with the staining samples of removed tissue from patients with DD initially treated with selective fasciectomy, and no difference was observed between the histological lesions in conventionally treated cases and those injected with CCH, which was consistent with other findings [23].

Selective fasciectomy after CCH injection does not report important difficulties in the operative act, as long as the CCH injection is performed according to the recommendations. There were no histological changes in the tissue after CCH injection.

### Electronic supplementary material

Below is the link to the electronic supplementary material.

Supplementary Material 1
