# Correction to: Long-term recurrence of Dupuytren ’s disease treated with clostridium histolitycum collagenase. Surgical treatment and anatomopathological study

**DOI:** 10.1007/s00402-024-05389-0

**Published:** 2024-06-19

**Authors:** C. Simón-Pérez, J. I. Rodríguez-Mateos, I. Aguado-Maestro, M. Alvarez-Quiñones, E. Simon-Perez, M. A. Martín-Ferrero

**Affiliations:** 1 Discipline of Orthopaedics, University of Valladolid, Valladolid, Spain; 2 Department of Plastic Surgery, Hospital Universitario Rio Hortega, Valladolid, Spain; 3 Department of Pathological Anatomy, Hospital Clínico Universitario, Valladolid, Spain; 4 DP Recoletas Felipe II Hospital C/ Felipe II, Valladolid, 9 47003 Spain; 5 Hospital Clínico Universitario, Avenida Ramón y Cajal, s/n, Valladolid, 47005 Spain

**Correction to: Archives of Orthopaedic and Trauma Surgery (2024) 144:2085–2091**

10.1007/s00402-024-05320-7

In the original version of this article, the given name and family name of author I. Aguado Maestro were incorrectly structured. The correct given name is I. and family name is Aguado-Maestro.

