# Supplementary material for: Long-term recurrence of Dupuytren’s disease treated with clostridium histolitycum collagenase. Surgical treatment and anatomopathological study
Source: Arch Orthop Trauma Surg. 2024 Apr 23;144(5):2085–91. doi: 10.1007/s00402-024-05320-7 (PMC11093830; doi:10.1007/s00402-024-05320-7)
Supplement: Supplementary file 1 — Supplementary Material 1 [file 402_2024_5320_MOESM1_ESM.docx]

***Long-term recurrence of Dupuytren's disease treated with Clostridium Histolitycum collagenase. Surgical treatment and anatomopathological study.***

**Conflicts of interest:** The authors declared no potential conflicts of interest with respect to the research, authorship, and/or publication of this article.

**Funding:** The authors declare that no funds, grants, or other support were received during the preparation of this manuscript.

**Financial statements:** The authors have no relevant financial or non-financial interests to disclose.

**Author Contributions:** All authors contributed to the study conception and design. Material preparation, data collection and analysis were performed by Clarisa Simon-Perez, Jose Ignacio Rodríguez-Mateos and Miguel Angel Martin-Ferrero the first draft of the manuscript was written by Clarisa Simon-Perez and all authors commented on previous versions of the manuscript. All authors read and approved the final manuscript.

All authors have contributed substantially to the conception or design of the work and to the acquisition, analysis or interpretation of data and have reviewed and approved the submitted version of the work.

All authors have agreed to be personally responsible for the author's contributions and to ensure that issues relating to the accuracy or completeness of any part of the work, even those in which the author was not personally involved, are properly investigated, resolved and resolved.

**Ethical approval details and details of informed consent:** Written informed consent was obtained from all patients, and ethical approval from the local Hospital Research Ethics Committee (University Hospital of Valladolid, Spain) was obtained. (PI 17-548- CINV 16-56).

**Consent to participate**: Informed consent was obtained from all individual participants included in the

study.

**Consent to publish:** The authors affirm that human research participants provided informed consent for

publication of the images in Figures

The datasets used and/or analysed during the current study available from the corresponding author on reasonable request.
